# Factors related to mortality due to progression of disease in patients with colon cancer in the presence of competing risks: a retrospective cohort study in the west of Iran 

**Published:** 2021

**Authors:** Ghodratollah Roshanaei, Malihe Safari, Javad Faradmal, Mohammad Abbasi, Salman Khazaei

**Affiliations:** 1 *Department of Biostatistics, School of Public Health, Modeling of Noncommunicable Diseases Research Canter, Hamadan University of Medical Sciences, Hamadan, Iran *; 2 *Department of Biostatistics, School of Public Health, Hamadan University of Medical Sciences, Hamadan, Iran*; 3 *Department of Internal Medicine, School of Medicine, Hamadan University of Medical Sciences, Hamadan, Iran*; 4 *Research Center for Health Sciences, Hamadan University of Medical Sciences, Hamadan, Iran*

**Keywords:** Competing risk, Colon cancer, Cause-specific risk, Death due to cancer progression

## Abstract

**Aim:**

This study aims to identify the risk factors of disease-related death in patients with colon cancer in the presence of competing risks.

**Background:**

Competing risk analysis is an effective method for identifying risk factors of death from disease, and the evidence related to the prognosis of death in patients with colon cancer in the country is rare.

**Methods:**

In this historical cohort study, the information of 196 patients with colon cancer who were referred to Imam Khomeini Clinic in Hamadan during the years 2003 to 2017 were examined. Death due to the progression of cancer was considered an interesting cause, and death related to other causes was considered a competing event. Predictors of death due to the progression of cancer were determined in the presence of competing risks. The cause-specific hazard regression model was used to determine the effects of covariates. Data was analyzed using R software vol. 3.4.3 and survival packages.

**Results:**

The mean (SD) age of patients was 57.1 (12.9) years, and 52.6% were male. The results of the multivariate cause-specific hazard regression model showed that the patient's age at the time of cancer diagnosis, T stage, stage of the disease and N stage had significant effects on the hazard of death due to cancer progression (*p*<0.05).

**Conclusion:**

In the presence of various causes of death, using the cause-specific hazard model to identify the risk factors of each cause separately can better support clinical decisions compared to other models.

## Introduction

 Colorectal cancer is the second and third common cancer in men and women, respectively, worldwide. According to estimates by the GLOBOCAN project in 2018, the incidence and mortality from colorectal cancer in both genders is 10.2 and 9.2 per 100,000 populations, respectively (1, 2). Except in the Caribbean, in other parts of the world, incidence and mortality rates are slightly higher in men than in women (3).

The survival rate of cancer can be an appropriate indicator to determine the overall impact of medical services on patient management. In recent years, the survival of these patients has improved dramatically through early identification and treatment with newer drugs. The five-year survival rate for colorectal cancer patients in the United States is 65% (4). The stage of the disease at the time of diagnosis and initiation of treatment is one of the predictors of survival time. The five-year survival rate of colorectal cancer in stages one and two varies from 80% to 90%, while the 5-year survival rate for the same patients in stages three and four of the disease are in the range of 60-71% and 13-18%, respectively (4).

Colorectal cancer includes those colon and rectal cancers caused by the uncontrolled growth of the lining of the colon and rectum, which increases with age (5). Studies show that the prognosis, clinicopathological factors, oncogenes, and tumor suppressor genes in colon and rectal cancers are different, and in general, the prognosis of colon cancer is better than that of rectal cancer. Therefore, it is important to investigate the determinants of survival in these patients separately by cancer type (6).

In classical survival studies, it is assumed that the event (usually death) occurs by a specific cause, which is the goal of the study, and that other causes are considered as censors. Usually in this approach, the Kaplan-Meier, Log-rank test, and Cox regression model or parametric models are used to analyze the data (7-9). However, some studies have presented situations in which the event may have occurred due to a variety of causes. In colon cancer, for example, death may be caused by the progression of the disease (interest cause) or other causes (competing cause).

Asghari Jafarabadi et al. evaluated the predictors of colon and rectal cancer death using the parametric competing risk model by assuming that the time-to-event followed Weibull distribution. In their study, age, body mass index (BMI: kg/m^2^), disease stage, and tumor grade were identified as predictors of survival in these patients (10). Storli et al. (2015) used the Cox model to evaluate the factors affecting the survival of colorectal cancer patients after surgery. They showed that age, sex, the ratio of high lymph nodes in the disease stage, and tumor stage were significant predictors of survival in these patients (11). Van Leewen et al. (2008) showed that increasing age is a factor affecting the survival of patients with colon cancer, but patient's gender is not a determining factor in their survival (12). Aquina et al. (2017) studied the effects of age-related differences in determining the 1-year rate and cause of death after surgery using competing risk methods in colon cancer patients (13). Holmes et al. (2017) evaluated the predictors of relapse in colon and rectum cancer patients in the presence of death as a competing risk (14).

Due to the efficacy of competing risk methods for analyzing survival data and the lack of evidence in this regard, the current study was designed to identify the risk factors of death due to progression of disease in colon cancer patients in the presence of competing risks. 

## Methods

**Introducing data**

In this retrospective cohort study, the data of 196 patients with colon cancer, referred to Imam Khomeini Specialized Clinic in Hamadan Province as a referral Center between 2003 and 2017, was examined. 

Patients' survival time was obtained from the difference between the date of death or censorship of patients from the date of their diagnosis. 

Inclusion criteria included a definitive pathological diagnosis of colon cancer and being native to Hamadan province. Lack of cooperation to complete survival information and major defects in the patient's medical record were considered as exclusion criteria.

During the study period, 86 patients died. The interesting outcome of this study was death due to cancer progression; 75 cancer patients died due to cause of interest, and 11 patients died from other causes considered competing risks. The surviving patients at the end of the study were considered as right-censored. Any defects or omissions in the medical records of patients in terms of demographic characteristics were completed as much as possible by phone call.

Patient information included gender (male, female), age at diagnosis (under 45, 46-65, and over 65 years), stage of cancer (first, second and third, fourth), metastasis (yes, no), chemoradiotherapy (yes, no), chemotherapy (yes, no), surgery (yes, no), T stage (T2, T3, T4, Tx), tumor grade (one (low grade), two (high grade) and three (with metastasis)), N stage (N0, N1 (1-2 involved glands), N2 (3-6 involved glands), N3 (more than 6 involved glands)), and tumor size (5 mm and less, more than 5 mm).

**Statistical Analysis**

The Kaplan-Meyer (KM) curve was used to calculate survival, and incidence rate (1-KM) was used without considering the competing risk. The cumulative incidence function was used to determine the incidence of death due to the interest cause and the competing cause. The cause-specific hazard regression model was used to determine the effect of risk factors on patient survival. R software vol. 3.6.1 and survival and cmprsk packages were used to analyze the data.

**Table 1:** Demographic and clinical characteristics of patients with colon cancer

Variable	Category	N(%)	Variable	Category	N(%)
Gender	male	103 (52.6)	Metastasis	yes	88 (44.9)
	female	93 (47.4)		no	108 (55.1)
Age at diagnosis (year)	Less than 50	56 (28.6)	Tumor size (cm)	<=5	125 (63.8)
	51-70	111 (56.6)		>5	37 (18.9)
	Greater than 70	29 (14.8)		unknown	34 (17.3)
Grade of tumor	good	79 (40.3)	N stage	N0	112 (57.1)
	moderate	101 (51.5)		N1	51 (26.0)
	poor	16 (8.2)		N2	33 (16.8)
Site of metastasis	liver	51 (57.5)	Tumor stage	II	89 (45.4)
	lung	7 (8.0)		III	52 (26.5)
	other	30(34.5)		IV	55 (28.1)
Chemotherapy	yes	168 (85.7)	Morphology of cancer	adenocarcinoma	192 (98.0)
	no	28 (14.3)		other	4 (2.0)
T stage	T2	25 (12.8)	Surgery	yes	180 (91.8)
	T3	136 (69.4)		no	16 (8.2)
	T4	23 (11.7)	Number of chemotherapy treatments	0	28 (14.3)
	Tx	12 (6.1)		<=5	69 (35.2)
BMI (kg/m^2^)	<18.5	37 (18.9)		>5	99 (50.5)
	18.5-25	120 (61.2)	Chemo-radiotherapy	yes	12 (6.1)
>25	39 (19.9)	no	184 (93.9)

## Results

In this study, the data of 196 colon cancer patients referred to Imam Khomeini Specialized Clinic in Hamadan province during the years 2003-2017 was examined. Mean patient age (SD) at diagnosis was 57.1 (12.9) years (range: 21-84 years), and 103 patients (52.6%) were male. The mean (SD) BMI of patients was 22.1 (3.6) kg/ m^2 ^(range: 12.2-31.4). Other patient characteristics are shown in Table 1.

To determine the cumulative incidence of death for various causes, the Kaplan-Meier curve and the cumulative incidence function were used in the presence of competing risks. As shown in Figure 1, the rate of outcome occurrence in the presence of competing risks was overestimated using the Kaplan-Meier method. Therefore, in the presence of competing risks, the actual rate of outcome occurrence should be estimated using the cumulative incidence function, which is shown in Table 2. Table 2 shows that 16% of patients died at the end of the first year due to cancer progression, and only 2% died due to other causes. By the end of the tenth year of follow-up in the present study, 50% of patients had died of cancer, and only 8% had died of other causes.

**Table 2:** Cumulative incidence rate of death due to progression of colon cancer and other causes of death

Time (months)	Cumulative incident of death due to cancer-related issues	Cumulative incident of death due to cancer-unrelated issues
12	0.16	0.02
24	0.29	0.02
36	0.37	0.02
48	0.39	0.04
60	0.46	0.04
120	0.50	0.08

The results of fitting the cause-specific hazard regression model showed that in the univariable analysis, age at diagnosis, surgery, T stage, disease stage, N stage, and number of chemotherapy sessions had significant impacts on death risk due to cancer progression (*p*<0.05). This study also simultaneously investigated the effects of significant variables on the hazard of death using a multivariate cause-specific hazard regression model.

The results in Table 3 show that the variables of T stage, disease stage, N stage, and number of chemotherapy treatments had a significant impact on the hazard of death due to cancer progression (*p*<0.05). Compared with patients in T2 stage, patients in T3, T4, and Tx had a 3.2, 6.8, and 15.4 times greater hazard, respectively. Patients with stage 3 and 4 tumors had a 3.64- and 21.2-fold risk compared with patients with stage 2 disease. The hazard of death in patients in stage N2 was 2.23 times that of patients in stage N0. Moreover, in patients who had received more than 5 chemotherapy treatments, the hazard of death was reduced by 62%.

## Discussion

The results of the present study showed that the main cause of death in patients with colon cancer is the progression of cancer itself, and competing causes play a small role in the occurrence of death in these patients. According to the results of the cause-specific hazard model, age at diagnosis, T stage, stage of disease, and T stage had significant effects on the risk of death due to cancer progression. In line with the results of other studies (15-17), the present study shows that the risk of death in patients over 70 years of age is significantly higher. In some studies, there was no significant difference between the outcome of the disease and the age of the patients (18, 19). The difference in results can be explained by the different study designs and the way of dealing with confounding variables.

Disease stage is always an important factor in disease outcome. The results of the present study showed that with increasing tumor stage, the risk of death increases significantly, which is in line with the results of other studies (15, 20). The results of a review study showed that the survival of these patients is highly dependent on the stage of the disease at the time of diagnosis; their 5-year survival varies from 90% in the early stages to 10% in the advanced metastatic stage (21). Survival analysis regarding colon cancer patients in the United States during the years 2009-2015 showed that the survival rate in these patients varied from 90% in those diagnosed in the early stages of the disease to 71% in the second stage and 14% in patients diagnosed with metastasis to other organs (22).

The results of the univariable regression model in this study showed that the risk of death in patients over the age of 70 years was more than twice that of patients under the age of 50. Invasive methods are less commonly used to treat people over the age of 70 who are diagnosed in advanced stages. These reasons can justify the higher survival of younger patients (12). Nipp et al. (2018) found that there are age and ethnic disparities in survival outcomes in cancers, and old age was associated with a lower likelihood of receiving chemotherapy, radiation, and/or surgery (23). In the study of Rodriguez et al. (2013), chemotherapy did not have a significant effect on increasing the survival of elderly patients (24). Aquina et al. studied 24,426 patients with colon cancer and found that older age and subsequent complications, such as sepsis, were associated with increased mortality from colon cancer as well as cardiovascular disease (13).

In the present study, number of chemotherapy treatments was associated with reduced mortality in patients. This may be due to the effects of other variables on increasing patient survival and, subsequently, increasing the number of chemotherapy treatments per patient. Furthermore, in patients whose tumor was removed by surgery, the risk of death was significantly lower in the univariable model. Chemotherapy is usually prescribed for patients whose cancer has metastasized to other organs. It can be argued that the increased survival in patients who have had surgery is due to the low grade of the tumor in these patients.

Compared to colon cancer, cancers in the rectum area have different biological characteristics, treatment methods, relapse patterns, and survival rates. Therefore, it seems that to better understand the prognosis of the disease, it is preferable to examine colon and rectum cancers separately. Akhoond et al. showed that the survival rate in colon cancer patients was about twice that of patients with rectal cancer, and the predictors of survival were different in the two groups of patients (25). Therefore, in the present study, only the predictors of survival in patients with colon cancer were examined.

The limitations of this study included the lack of access to some clinical information related to the participants as well as the lack of a cause of death for some patients due to the retrospective nature of this study. Moreover, failure to revisit some of the patients in the early stages of the disease resulting in a lack of access to sufficient information from them as well as changes in telephone numbers or patients' addresses for tracking their survival or death were other limitations. The strengths of this study include the fact that information was gathered from Imam Khomeini Clinic in Hamadan province as the only referral center for colorectal cancer patients in the province, and that all treatment procedures were performed by an oncologist.

After fitting the multivariate regression model, the variables of T stage, disease stage, N stage, and number of chemotherapy treatments had significant effects on the hazard of patient death. In the presence of various causes of death, to identify the risk factors for each cause separately, the use of a cause-specific hazard model can provide better support for clinical decisions compared to other models.

**Table 3:** Assessment of risk factors on hazard of death due to progression of cancer in patients with colon cancer using uni- and multi-variate cause-specific hazard regression model

Variable	Category	HR (95%CI)	Adjusted HR (95%CI)
Gender	male	1	-
	female	1.06 (0.67, 1.66)	-
Age at diagnosis (year)*	Less than 50	1	1
	51-70	0.71 (0.42, 1.19)	0.84 (0.49, 1.44)
	Greater than 70	2.1 (1.1, 3.98)*	1.1 (0.55, 2.17)
Grade of tumor	good	1	-
	moderate	1.27 (0.79, 2.07)	-
	poor	1.36 (0.84, 4.12)	-
Chemotherapy	yes	1	-
	no	2.24 (0.9, 5.6)	-
Chemo- radiotherapy	yes		-
	no	1.13 (0.49, 1.6)	-
Surgery	no	1	-
	yes	0.2 (0.11, 0.38)	0.92 (0.44, 1.94)
T stage *	T2	1	1
	T3	2.7 (0.98, 7.5)*	3.2 (1.3, 20.7)*
	T4	5.7 (1.9, 17.1)*	6.8 (2.9-27.84)*
	Tx	14.9 (4.7, 47.67)*	15.4 (3.2, 55.5)*
Tumor size (cm)	<=5	1	-
	>5	1.1 (0.6, 2.02)	-
BMI (kg/m^2^)	<18.5	1	-
	18.5-25	0.58 (0.33, 1.02)	-
	>25	0.79 (0.41, 1.52)	-
Tumor stage*	II	1	1
	III	4.64 (2.1, 10.6)*	3.64 (1.23, 10.8)*
	IV	22.2 (10.2, 47.9)*	21.2 (8.3, 53.9)*
N stage*	N0	1	
	N1	2.11 (1.2, 3.75)*	1.1 (0.53, 2.31)
	N2	7.33 (4.2, 12.8)*	2.23 (1.11, 4.45)*
Number of chemotherapy	5 ≤	1	1
5 >	0.37 (0.23, 0.59)*	0.38 (0.13, 0.61)

*Significant at 5% level
